# Association of Physical Activity With Telomere Length Among Elderly Adults - The Oulu Cohort 1945

**DOI:** 10.3389/fphys.2019.00444

**Published:** 2019-04-24

**Authors:** Ville Stenbäck, Shivaprakash Jagalur Mutt, Juhani Leppäluoto, Dominique D. Gagnon, Kari A. Mäkelä, Jari Jokelainen, Sirkka Keinänen-Kiukaanniemi, Karl-Heinz Herzig

**Affiliations:** ^1^Research Unit of Biomedicine, Department of Physiology and Biocenter of Oulu, University of Oulu, Oulu, Finland; ^2^Medical Research Center, University of Oulu, Oulu, Finland; ^3^Laboratory of Environmental Exercise Physiology, School of Human Kinetics, Laurentian University, Sudbury, ON, Canada; ^4^Centre for Research in Occupational Safety and Health, Laurentian University, Sudbury, ON, Canada; ^5^Center for Life Course Health Research, Faculty of Medicine, University of Oulu, Oulu, Finland; ^6^Unit of General Practice, Oulu University Hospital, Oulu, Finland; ^7^Department of Gastroenterology and Metabolism, Poznan University of Medical Sciences, Poznań, Poland

**Keywords:** physical activity, elderly, telomeres, objective measurements, step counts, questionnaires

## Abstract

**Introduction:** Physical activity (PA) has been associated with telomere shortening. The association of PA intensity or volume with telomere length (TL) is nonetheless unclear. The aim of our study was to investigate the associations of exercise intensity and volume with TL in elderly adults from Northern Finland (65° latitude North).

**Methods:** Seven hundred elderly subjects born in 1945 in the Oulu region were investigated. PA was measured during a 2-week period with a wrist-worn accelerometer. In addition, a questionnaire was used to assess sedentary time and to achieve a longitudinal PA history and intensity. Relative telomere lengths (RTL) were determined from frozen whole blood samples using a qPCR-based method.

**Results:** Relative telomere lengths were significantly longer in women than men and negatively correlated with age in both genders (men *r* = -0.210, *p* = 0.000, women *r* = -0.174, and *p* = 0.000). During the 2-week study period, women took more steps than men (*p* = 0.001), but the association between steps and RTL was only seen in men (*p* = 0.05). Total steps taken (*r* = 0.202 and *p* = 0.04) and sedentary time (*r* = -0.247 and *p* = 0.007) significantly correlated with RTLs in 70-year old subjects. Moderate PA was associated with RTL in subjects with the highest quartile of moderate PA compared to the three lower quartiles (*p*-values: 0.023 between 4th and 1st, 0.04 between 4th and 2nd, and 0.027 between 4th and 3rd) in the 70-year old subjects.

**Conclusion:** Women had longer RTL and a higher step count compared to men. However, exercise volume and RTL correlated positively only in men. Surprisingly, age correlated negatively with RTL already within an age difference of 2 years. This suggests that telomere attrition rate may accelerate in older age. Moderate physical activity at the time of study was associated with RTL.

## Introduction

Telomeres are looped structures located at the end of chromosomes, protecting our genomic DNA from degradation. Telomeric DNA consists of repetitive sequences of TTAGGG, common to all mammals (Aubert and Lansdorp, 2008). Double-stranded structure changes into single-stranded structure, creating a 3′ overhang in the G-rich strand, which is a principal feature in loop formation, hiding the chromosomal ends from the DNA damage repair machinery. Due to the properties of DNA replication, DNA synthesis cannot proceed to the end of the chain (end-replication problem), thus shortening of the telomere with each cell division by approximately 50–100 bp occurs (Sanders and Newman, 2013). In addition to telomeric DNA, the six subunit protein complex shelterin is needed for telomere structure and function (Podlevsky and Chen, 2012). Different subunits interact with DNA and telomerase holoenzyme. Telomerase adds TTAGGG repeats onto the chromosomal ends and thus is responsible for telomere length (TL). Telomerase is active in germ and stem cells, while its activity diminishes in somatic tissues, leading to telomere shortening with each cell division (Hayflick limit). At least a 400 bp of the telomeric repeat sequence is needed for maintaining a functional telomere, but experiments with cancer cell lines have demonstrated that TL less than 1 kb is sufficient to induce senescence (de Lange, 2009). Human TLs are between 10 and 15 kb at birth, and then gradually decline.

Telomere length at birth is similar in both genders, but women have longer telomeres later in life (Seifarth et al., 2012). Estrogen and higher compatibility between mitochondrial and genomic DNA have been associated with higher TL in women. TL has been shown to be hereditary from the paternal side (Nordfjäll et al., 2005). Furthermore, previous work has shown that high stress levels (both psychological and oxidative, determined via the 10-item Perceived Stress Scale and isoprostanes per milligram of creatinine/vitamin E) are associated with shortened TL (Epel et al., 2004). Seventy-five minutes of vigorous exercise weekly was found to be associated with longer telomeres when experiencing psychological stress (The 10-item Perceived Stress Scale) (Puterman et al., 2010). These findings suggest a complex network influencing the maintenance and integrity of telomeres, which includes genetic, lifestyle, psychological and physiological factors.

Several studies have shown that low PA is associated with telomere shortening (Ludlow et al., 2008; Puterman et al., 2010; Ludlow and Roth, 2011; Bojesen, 2013; Weischer et al., 2014; Shadyab et al., 2017a,b; Williams et al., 2017). Tucker (2017) demonstrated that sedentary people were 9 years pre-aged on the cellular levels (based on shorter TL) compared to people in the high PA activity group. Conversely, Savela et al. (2013) reported that subjects exercising with moderate intensity (MPA) had the longest telomeres. At the cellular level an age difference of 4 to 6 years was observed between those of moderate compared to those of low intensity PA. Maximal oxygen uptake (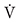
O_2_ max) has been shown to positively correlate with TL (LaRocca et al., 2010; Østhus et al., 2012). Interestingly, however, in extreme endurance athletes (e.g., marathon runners), TL is similar to sedentary subjects, suggesting that excessive training might be harmful (Mathur et al., 2013). Training hours and years of practice at a professional level correlated negatively with TL in professional endurance runners (Rae et al., 2010). The same association was observed in competitive powerlifters; the TL in their *vastus lateralis* correlated inversely with the subject’s record in squat and deadlift (Kadi et al., 2008). These findings suggest an inverted U-shaped relationship between PA intensity and TL, with both high and low PA levels associated with shortened TL. Shadyab et al. (2017a) showed that greater amounts of moderate-to-vigorous PA were associated with longer telomeres in elderly women. Among less physically active older women, sedentary time was associated with shorter TL (Shadyab et al., 2017b). Shorter telomeres are associated with limitations in physical functioning compared to subjects with long telomeres in elderly European populations (Rojas et al., 2018).

In addition, production of reactive oxygen species (ROS) contributes to increased DNA damage, apoptosis and senescence (Kawanishi and Oikawa, 2004). Especially, ROS have been shown to influence the central 5′-GGG-3′ guanine segment, abundant in telomeric DNA (Bojesen, 2013; Arsenis et al., 2017). Importantly, regular PA has been showed to reduce ROS levels (He et al., 2016). Based on the studies mentioned above, the relationship between PA amounts and intensities and TL is still unclear, especially in the older age groups. The aim of our study was to assess the associations of volume and intensity of PA with TL among older adults in a cross-sectional study in the Oulu cohort 1945 from Northern Finland. We hypothesized that higher amounts of PA would be associated with longer TL.

## Materials and Methods

### Study Population

**FIGURE 1:**
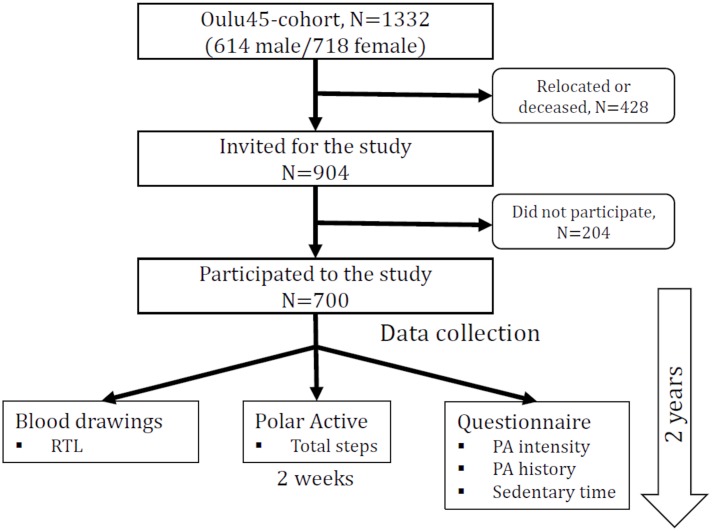
Flow chart of the study.

The study population was based on a health survey conducted in 2002 among all persons born in 1945 and living in the City of Oulu, Finland (120 000 inhabitants, 65°01′ N, 25°28′ E) (Juuti et al., 2008). 904 of those were invited for a follow up study during the years 2013–2015 (Figure 1). 204 declined to participate. The data collection took place over 2 years, resulting in a maximum age difference of 2 years within the study population. The study was approved by the ethical committee of the Northern Ostrobothnia Hospital District and has been carried out according to the National legislation and guidelines and the declaration of Helsinki. All subjects gave written informed consent in accordance with the Declaration of Helsinki. The data collection has been previously described (Metsämarttila et al., 2018). Covariates presented in Table 1 were collected during visits in 2013–2015. Age, gender, education, smoking and hypertension medication usage were assessed by a questionnaire. Weight, height, waist circumference and blood pressure were measured and blood samples drawn by a licensed nurse. Body mass index (BMI) was calculated as weight in kilograms divided by height meters squared. Cholesterol, triglycerides, C-reactive protein (CRP), glycated hemoglobin A1c (HbA1c), fasting glucose, fasting insulin, and relative telomere length (RTL) were analyzed from the blood samples. Homeostatic model assessment of insulin resistance (HOMA-IR) was calculated according to the formula: fasting insulin (μl/L) x fasting glucose (nmol/L) / 22.5. The metabolic syndrome (MetSyn) was defined according the new International Diabetes Foundation definition (Alberti et al., 2005).

**Table 1:** Anthropological and biochemical characteristics of the study population.

		Total	Men	Women	*p*-value^a^	*p*-value^b^
Number of subjects		700	296 (42.3%)	404 (57.7%)	–	0.0975
Age (years)		68.9 ± 0.6	68.9 ± 0.5	68.9 ± 0.6	0.595	<0.0001*
Education	Level	–	–	–	–	0.468
	1	94 (13.4%)	40 (13.6%)	54 (13.2%)	–	–
	2	167 (23.9%)	76 (25.7%)	91 (22.7%)	–	–
	3	155 (22.1%)	59 (19.9%)	96 (23.7%)	–	–
	4	159 (22.7%)	54 (18.0%)	105 (26.0%)	–	–
	5	125 (17.9%)	67 (22.8%)	58 (14.5%)	–	–
Smoker		90 (12.8%)	41 (15.3%)	44 (11.0%)	0.105	0.3294
Alcohol consumption (g/day)		1.9 ± 4.6	2.9 ± 4.7	1.2 ± 4.5	<0.0001*	–
BMI (kg/m2)		27.68 ± 4.7	28.0 ± 4.3	27.5 ± 5.1	0.164	0.417
Waist (cm)		94.2 ± 13.7	100.0 ± 12.1	90.0 ± 13.3	<0.0001*	0.458
SP (mmHg)		143.9 ± 17.5	145.7 ± 16.31	142.6 ± 18.2	0.018*	0.681
DP (mmHg)		85.5 ± 9.6	86.1 ± 9.3	85.1 ± 9.8	0.178	0.085
T/S Ratio		0.8 ± 0.3	0.8 ± 0.2	0.8 ± 0.3	0.1056	–
Total cholesterol (mmol/l)		5.3 ± 1.2	4.9 ± 1.1	5.6 ± 1.3	<0.0001*	0.776
HDL cholesterol (mmol/l)		1.7 ± 0.5	1.5 ± 0.4	1.8 ± 0.4	<0.0001*	0.626
Triglycerides (mmol/l)		1.3 ± 0.8	1.3 ± 1.0	1.2 ± 0.6	0.123	0.237
CRP (mg/l)		3.5 ± 9.3	3.2 ± 7.0	3.2 ± 10.9	0.453	0.728
HbA1c (%)		5.9 ± 0.5	5.9 ± 0.6	5.9 ± 0.6	0.9897	0.008*
HOMA-IR		1.9 ± 1.4	2.0 ± 1.5	1.8 ± 1.2	0.0982	0.328
MetSyn		362 (51.7%)	131 (44.1%)	226 (55.9%)	0.2486	–
Hypertension medication		366 (52.3%)	128 (43.3%)	229 (56.7%)	0.046*	0.198


*Means ± standard deviations, numbers of subjects and percent of subjects n(%). a = Significance of the difference between the genders. b = significance of the Pearson correlation between each variable and RTL (T/S Ratio). SP: systolic pressure, DP: diastolic pressure. ^∗^*p*-value < 0.05.*

### Activity Measurements

We have used two different approaches to determine the level of physical activity. The detailed description and validation of both objective and subjective physical activity measurements was recently reported (Niemelä et al., 2019). The subjects wore the same wrist-worn accelerometer (Polar Active, Polar Electro, Finland) for 2 weeks to record their habitual physical activity. The device recorded the total amount of steps. In addition, subjects filled out a questionnaire which included questions of their current and past PA frequency, intensity and sedentary time. The intensity and frequency were assessed at four time-points (ages of 15, 30, 50, and current age). At these points light intensity (LPA), MPA and vigorous intensity PA (VPA) was determined. LPA consisted of light cycling, walking, gardening, indoors chores and motorbiking. Brisk walking, calm swimming, ice skating, wood or water carrying, brisk cycling, gymnastics at home, and horseback riding was characterized as MPA. VPA included climbing stairs, rowing/cycling/swimming fast, skiing, shoveling/shoveling snow, and brisk walking in the swamp. Subjects were asked to fill in separately, how many times they did these three types of exercise in a week at each point of their lives which were then divided into quartiles for analysis. The quartiles were defined according to how many times a week the subjects engaged in different activities (LPA, MPA, and VPA separately). Time spent sitting in different situations (e.g., at work, commuting, and watching TV) were used to compose the total daily sedentary time during a normal weekday (Supplementary Table S1).

### Relative Telomere Length Determination

DNA was isolated from frozen whole blood samples using the Nucleospin DNA blood kits (MACHEREY-NAGEL GmbH & Co., KG, Germany) according to manufacturer’s instructions with minor modifications in protocol such as increasing lysis incubation time from 10 to 30 min. RTL was determined with qPCR using Cawthon’s monochrome multiplex method (Cawthon, 2002, 2009). Briefly, the 2 μl DNA samples were amplified for 40 cycles using either telomere or β-globin primers and the FastStart Universal SYBR Green Master reagent (Roche) in 20 μL final reaction volume. Reactions were run using telomere primers and beta-globin (SCG: single copy gene) primers on ABI 7300 real-time PCR system (Applied Biosystems, CA, United States) according to the following conditions: for telomere 95°C for 10 min, 2 cycles of 95°C for 15 s, 49°C for 15 s and 40 cycles of 95°C for 15 s, 60°C for 15 s, 70°C for 1 min and for β-globin 95°C for 10 min, 40 cycles of 95°C for 15 s, 60°C for 1 min followed by a dissociation (or melt) curve for PCR product verification. The *Ct*-values from both telomere (T) and SCG (S) expression were used to calculate the RTL (T/S ratio) by using the 2^-(ΔCt1-ΔCt2)^ = 2^-ΔΔCt^ and will be referred as RTL in the following.

### Statistics

The RTL values were used in all the statistical testing. The analysis was performed between RTL, and PA intensity levels among the 68-, 69-, and 70-year age groups as well as the four time points (15, 30, 50, current) to assess associations. Subjects were also divided to quartiles based on the PA volume (number of steps) and RTL was compared between quartiles. We used the Kruskall–Wallis test for comparison of continuous variables between groups. For group comparison, including gender and PA frequency and volume quartiles Mann–Whitney *U*-test was used. Correlations between variables (RTL, PA volume, and sedentary time) were assessed using Pearson correlation. Multiple linear regression models were utilized to evaluate the associations of steps with log-transformed RTL. The model was adjusted for age and potential confounder including education (Adler et al., 2013), alcohol consumption (Wang et al., 2017), smoking, BMI (Weischer et al., 2014), triglycerides, high-density lipoprotein (HDL) (Révész et al., 2014), and type 2 diabetes (Salpea et al., 2010). *P*-value of or less than 0.05 was considered significant. Presented numbers are mean ± standard deviation. Statistical analyses were done using IBM SPSS Statistics 21 and SAS 9.3.

## Results

### Age and Gender

**FIGURE 2:**
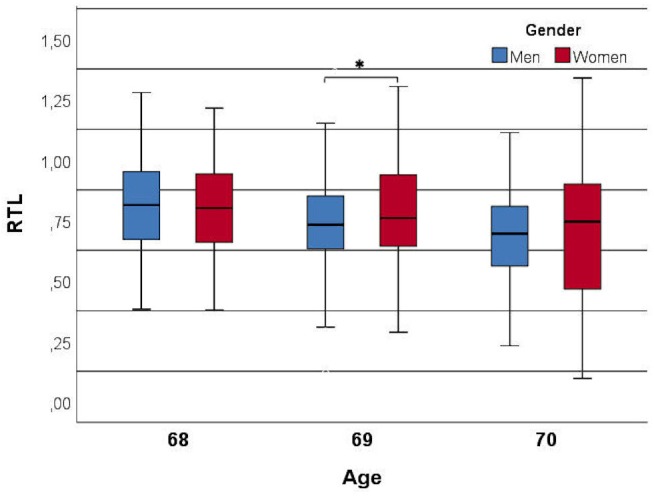
Relationship between relative telomere length (RTL) and age divided by genders. Age is negatively correlated with RTL (*r* = –0.185 and *p* = 0.0001) and a gender difference was observed in the 69-year old subjects (*p* = 0.031). ^∗^ indicates the significance.

We examined 700 subjects, 296 males and 404 females from the Oulu cohort (Table 1). Age was negatively correlated with RTL (*r* = -0.185 and *p* = 0.0001) within the study population and the correlation was stronger in men than in women (*r* = -0.210 and *r* = -0.174, respectively) (Figure 2). Since age was significantly associated with telomere length (TL) (Table 1), we divided the population into three groups based on age for further analysis. Interestingly, the age-stratified gender difference was observed in the 69-year old group only (*N* = 397) with women having longer RTL than men (*p* = 0.037). This difference was not observed in neither the 68- (*n* = 180) nor the 70-year old group (*n* = 123; *p* = 0.678 and *p* = 0.702, respectively).

### Exercise Volume

**FIGURE 3:**
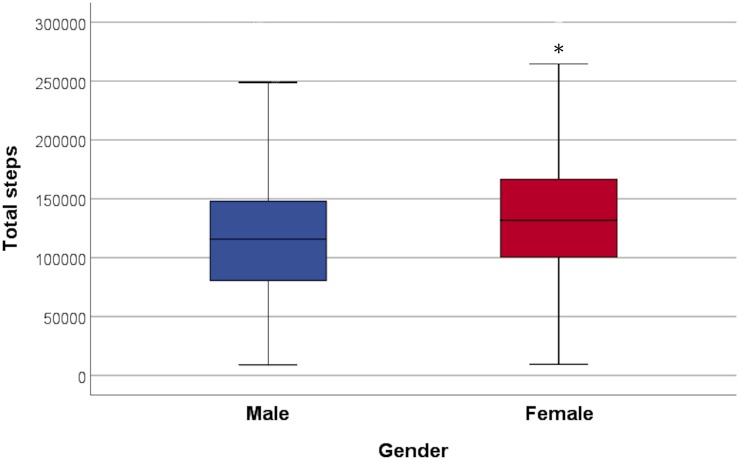
Total steps taken during the 2-week study period in both genders. Females took significantly more steps than males (*p* = 0.0001). ^∗^ indicates the significance.

**FIGURE 4:**
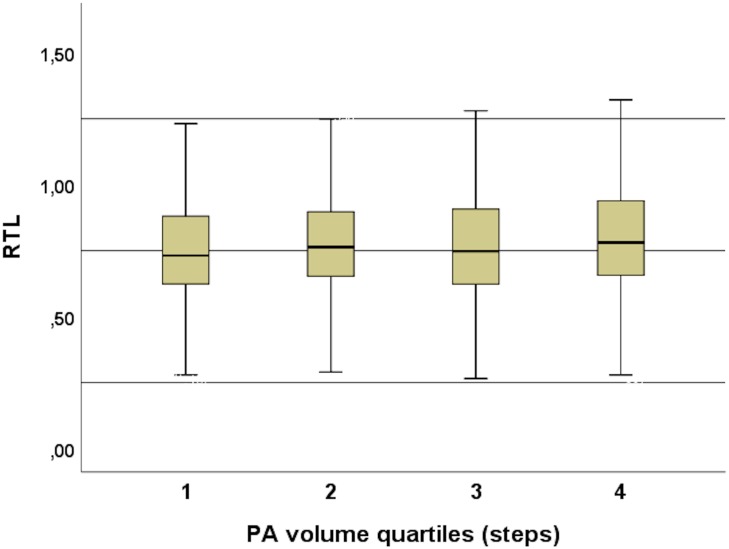
PA volume quartiles and RTL. No significant differences were observed between the quartiles (*p*-values > 0.05).

During the 2-week study period, the subjects took 131799 ± 58535 steps in total and women took significantly more steps than men (women 138479 ± 57557, men 122533 ± 58721, and *p* = 0.0001) (Figure 3), but the positive correlation between total amount of steps and RTL was significant only in men (*p* = 0.05). The association between steps and RTL in men remained significant after adjustment for age, but not with other confounders (Table 2). In the 70-year old group, sedentary time was negatively correlated with RTL (*r* = -0.247 and *p* = 0.007), but the total number of steps taken during the 2-week study period was positively correlated with RTL (*r* = 0.202 and *p* = 0.04). However, such correlations were not observed with neither the 68- nor the 69-year old group, nor with the whole study population. We also divided the subjects into quartiles using the mean daily steps and compared the RTL between quartiles (Figure 4). No significant differences were observed (*p*-values > 0.05).

**Table 2:** Association of mean steps taken and RTL in men (*N* = 296).

	Model 1			Model 2			Model 3			Model 4		
Variable	B	SE	*p*-value	B	SE	*p*-value	B	SE	*p*-value	B	SE	*p*-value
Intercept	-1.034	0.347	0.003	-0.884	0.388	0.024	-1.113	0.501	0.027	-1.113	0.502	0.028
Age	-0.130	0.037	0.000	-0.133	0.040	0.001	-0.131	0.040	0.001	-0.131	0.040	0.001
Mean steps	0.077	0.039	0.049	0.064	0.043	0.143	0.074	0.048	0.126	0.075	0.049	0.124
Education 1				-0.055	0.056	0.330	-0.052	0.057	0.362	-0.052	0.057	0.357
Education 2				0.008	0.049	0.869	0.010	0.050	0.835	0.010	0.050	0.849
Alcohol				-0.006	0.005	0.247	-0.007	0.006	0.222	-0.007	0.006	0.221
Smoker				-0.027	0.060	0.651	-0.021	0.061	0.728	-0.022	0.061	0.716
Triglycerides							-0.011	0.021	0.607	-0.011	0.021	0.602
HDL							0.006	0.059	0.912	0.008	0.059	0.897
BMI							0.005	0.006	0.405	0.004	0.006	0.444
Type 2 diabetes										0.010	0.052	0.841


*B = beta estimate, SE = standard error. Model 1 includes RTL, mean steps and age; Model 2 includes Model 1 variables and alcohol consumption, smoking status and education; Model 3 includes variables from Models 1 and 2 and triglycerides, HDL, and BMI. Model 4 includes variables from models 1–3 and type 2 diabetes. Education 1: level of basic education. Education 2: level of professional/vocational education.*

### Exercise Intensity by Questionnaire

**FIGURE 5:**
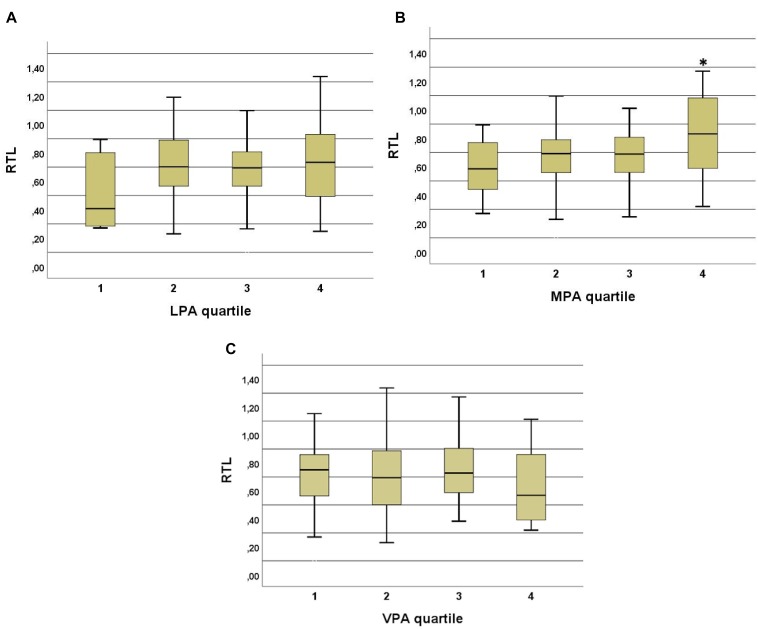
**(A)** Low intensity physical activity (LPA) quartiles and RTL in 70-year old subjects. No significant correlations were observed between the quartiles but there is a trend to shorter RTL in the quartile with the lowest physical activity (at least 15 min once for a week or less). **(B)** Moderate intensity physical activity (MPA) quartiles and RTL in 70-year old subjects. A significant difference was observed between the highest quartile and three lower quartiles. *p*-values are 0.023 between 4th and 1st, 0.04 between 4th and 2nd, and 0.027 between 4th and 3rd quartiles. People in the highest quartile engage in MPA 5 times or more in a week at least for 15 min at the time. ^∗^ indicates the significance. **(C)** Vigorous physical activity (VPA) quartiles and RTL in 70-year old subjects. No significant differences were observed between the quartiles. In the highest quartile the RTL is slightly but not significantly lower than in the three other quartiles.

The highest quartile (4th) of MPA (at age 68–70) resulted in significantly different RTL compared to those subjects within the three lowest MPA quartiles (Figure 5). The subjects in the highest quartile took significantly more daily steps on average in comparison to those in the other 3 quartiles (*p* < 0.001). This was observed among the 70-year old subjects but not in the 68- or 69-year old age groups. Earlier physical activities at the age of 15, 30, and 50-years old did not reveal any significant differences in current RTL. VPA quartiles at age of 50 and daily average steps at age of 68 to 70 were significantly different (*p* = 0.001).

## Discussion

In this study, we investigated the associations of self-reported and objective PA with RTL among elderly adults from the Oulu Birth Cohort 1945. The small age difference in the subjects was negatively correlated with RTL (*r* = -0.185 and *p* = 0.0001) with a stronger correlation in men (*r* = -0.210 and *r* = -0.174, respectively). Telomeres are known to have a high inter-variability, based on epigenetics and telomerase preferences (Nordfjäll et al., 2009). In our cohort, women had higher RTL than men after adjustment for age (*p* = 0.037), as previously described (Weischer et al., 2014). This phenomenon could be explained in part via traditional gender roles still existing in this age group in Finland. For example, men are normally doing the more physically demanding chores (snow shoveling, wood cutting, and renovation) than women who engage more often in everyday tasks such as cleaning and cooking. In addition, women are normally more aware of their health with better nutrition and vitamin supplementation (Radimer et al., 2004; Undén et al., 2008).

Objectively measured PA during a 2-week period with wrist-worn accelerometers was positively correlated with RTL in men at 68–70 years (*r* = 0.118 and *p* = 0.05) and in both genders at 70 years (*r* = 0.202 and *p* = 0.04). The association between steps and RTL in men remained after adjustment for age (model 1) but did not persist after adjustment for other potential confounder (alcohol consumption, smoking, education, triglycerides, HDL, BMI, and type 2 diabetes) (Table 2). Women took more steps during the study period, yet the positive association of exercise with RTL was only seen in men. In the 70-year old subjects, sedentary time was negatively correlated with RTL (*r* = -0.247 and *p* = 0.007). Previous studies are in line with our findings. A similar association was observed by Shadyab et al. (2017b) in 1,481 elderly women (aged 79.2 ± 6.7), with a shorter TL in less active subjects (higher sedentary time). In the same subjects, higher amounts of moderate to vigorous PA were associated with longer TL (Shadyab et al., 2017a). In addition, Weischer et al., 2014 observed a significant association between physical inactivity and shorter TL in subjects aged 47 to 76 years (*N* = 4,576 both sexes). These findings suggest that higher levels of physical activity are associated with longer TL (Ludlow and Roth, 2011; Bojesen, 2013). Puterman et al. (2010) studied 63 women (aged 61.9 ± 6.5) and observed that subjects with higher PA levels had less psychological stress (validated stress questionnaire) and longer telomeres. In our cohort, we did not observe an inverted U-shaped relationship between exercise volume and RTL (Figure 4), which was reported in other studies involving athletes (Kadi et al., 2008; Ludlow et al., 2008; Rae et al., 2010; Mathur et al., 2013). This finding suggests that the relationship seen in athletes might not be applicable to general population and certainly not to the elderly with their usual lack of larger amounts of vigorous exercise. Laine et al. (2015) found no differences between the RTL of former Finnish male athletes and their non-athlete counterparts (*N* = 599), that were similar in age to our study (athletes 72.7 ± 6.1 and controls 71.6 ± 5.6).

We found that only moderate PA was significantly associated with RTL. The 70-year old subjects, who engaged in MPA 5 or more times in week at least 15 min at a time had higher RTL than those subjects, who did less. In accordance with this, subjects in the lowest LPA quartile and highest VPA quartile had lower RTL, but the differences between other quartiles were not significant (Figure 5), indicating that MPA is more strongly associated in terms of longer TL in elderly subjects (Ludlow and Roth, 2011). The associations of previous PA at the ages of 15, 30, and 50 with RTL at the age of 70 were not significant. Interestingly, the reported level of VPA at age 50 correlated with the number of steps taken during the measurement period. These data suggest that subjects in this study may have developed PA habits in midlife that continued into old age.

### Strengths and Weaknesses of Our Study

This study has several strengths. All participants of our study were born in the same year and lived in the same region. They shared similar conditions in terms of environment, lifestyle and healthcare throughout their lives and have the same ethnic background. Moreover, PA presented in this study was measured objectively. This study also has several limitations. Wrist-worn accelerometers have been shown to overestimate step counts compared to waist-worn accelerometers, which makes it more difficult to compare these results with other studies with different accelerometers (Lee et al., 2015). In contrast, a strong association between self-reported leisure time physical activity and accelerometer-based step counts (Polar Active) in Northern Finland Birth Cohort 1966 study has recently been reported (Niemelä et al., 2019). Furthermore, the questionnaire has been only recently validated at age 46 in a similar study population in Northern Finland. Our elderly adult participants are considered as “survivors” of that age cohort. Since they were 68–70-years old at the data collection time, we hypothesize that the people with the worst lifestyle and lowest levels of activity did not participate in the study or were deceased. We cannot exclude recall bias, since a self-reported questionnaire was used to obtain data. Further linear regression modeling was not performed since the number of subjects was too small for further multiple linear regression modeling.

## Conclusion

In conclusion, we found, in an elderly cohort, born in 1945 in Northern Finland (latitude 65° North), that women had longer RTL and performed a higher volume of exercise compared to men. In addition, exercise volume and RTL were correlated positively in men but not in women. Age correlated negatively with RTL even with the age difference of only 2 years. We did not observe an inverted U-shaped relationship between PA volume and RTL. Moderate physical activity at the time of the study was positively associated with RTL.

## Ethics Statement

The study was approved by the ethical committee of the Northern Ostrobothnia Hospital District and has been carried out according to the National legislation and guidelines and the Declaration of Helsinki. Written informed consent was given by all subjects.

## Author Contributions

K-HH, JL, DG, and SK-K designed the study and provided the funding. VS, KM, and SM did the telomere analysis in the cohort subjects. VS and JJ did the data analysis. VS wrote the first draft of the manuscript. All authors contributed to writing of the manuscript.

## Conflict of Interest Statement

The authors declare that the research was conducted in the absence of any commercial or financial relationships that could be construed as a potential conflict of interest.
