# Supplementary material for: Association of Physical Activity With Telomere Length Among Elderly Adults - The Oulu Cohort 1945
Source: Front Physiol. 2019 Apr 24;10:444. doi: 10.3389/fphys.2019.00444 (PMC6499171; doi:10.3389/fphys.2019.00444)
Supplement: Supplementary file 1 [file Data_Sheet_1.docx]

**Supplementary Table 1.**

| **A** |  |  |  |  |
| --- | --- | --- | --- | --- |
| **How many times a week have you exercised at least for 15 minutes at following ages? LPA/MPA/VPA** | | | | |
|  | **less than once a week** | **1-2 times a week** | **3-4 times a week** | **at least 5 times a week** |
| **68-70** | **1** | **2** | **3** | **4** |
|  |  |  |  |  |
| **B** |  |  |  |  |
| **How many hours do you sit during a weekday in average? (put 0 if none)** | | | |  |
|  |  | **Hours** | **Minutes** |  |
| **At the office or similar** | |  |  |  |
| **At home reading** | |  |  |  |
| **At home watching TV** | |  |  |  |
| **At home in front of computer** | |  |  |  |
| **In a vehicle (car, bus, etc.)** | |  |  |  |
| **Other** |  |  |  |  |

The questionnaire parts were asked about physical activity and sedentary time. A, how many times a week the subject exercised at least 15 minutes at age of 68-70 (time of data collection). This question was answered separately for LPA, MPA and VPA. The answers were used to create quartiles and compare telomere lengths. B, subjects were asked how many hours they sit during a weekday in average.
